# T-Lymphocyte Responses to Intestinally Absorbed Antigens Can Contribute to Adipose Tissue Inflammation and Glucose Intolerance during High Fat Feeding

**DOI:** 10.1371/journal.pone.0013951

**Published:** 2010-11-11

**Authors:** Yuehui Wang, Jianing Li, Lihua Tang, Yu Wang, Richard Charnigo, Willem de Villiers, Erik Eckhardt

**Affiliations:** 1 Graduate Center for Nutritional Sciences and Internal Medicine Department, University of Kentucky, Lexington, Kentucky, United States of America; 2 Department of Biostatistics, University of Kentucky, Lexington, Kentucky, United States of America; New York University, United States of America

## Abstract

**Background:**

Obesity is associated with inflammation of visceral adipose tissues, which increases the risk for insulin resistance. Animal models suggest that T-lymphocyte infiltration is an important early step, although it is unclear why these cells are attracted. We have recently demonstrated that dietary triglycerides, major components of high fat diets, promote intestinal absorption of a protein antigen (ovalbumin, “OVA”). The antigen was partly transported on chylomicrons, which are prominently cleared in adipose tissues. We hypothesized that intestinally absorbed gut antigens may cause T-lymphocyte associated inflammation in adipose tissue.

**Methodology/Principal Findings:**

Triglyceride absorption promoted intestinal absorption of OVA into adipose tissue, in a chylomicron-dependent manner. Absorption tended to be higher in mesenteric than subcutaneous adipose tissue, and was lowest in gonadal tissue. OVA immunoreactivity was detected in stromal vascular cells, including endothelial cells. In OVA-sensitized mice, OVA feeding caused marked accumulation of CD3+ and osteopontin+ cells in mesenteric adipose tissue. The accumulating T-lymphocytes were mainly CD4+. As expected, high-fat (60% kCal) diets promoted mesenteric adipose tissue inflammation compared to low-fat diets (10% Kcal), as reflected by increased expression of osteopontin and interferon-gamma. Immune responses to dietary OVA further increased diet-induced osteopontin and interferon-gamma expression in mesenteric adipose. Inflammatory gene expression in subcutaneous tissue did not respond significantly to OVA or dietary fat content. Lastly, whereas OVA responses did not significantly affect bodyweight or adiposity, they significantly impaired glucose tolerance.

**Conclusions/Significance:**

Our results suggest that loss or lack of immunological tolerance to intestinally absorbed T-lymphocyte antigens can contribute to mesenteric adipose tissue inflammation and defective glucose metabolism during high-fat dieting.

## Introduction

Obesity is an important component of the metabolic syndrome [1] and represents a strong risk factor for cardiovascular disease [2]. Obesity is associated with inflammatory responses in adipose tissues [3], and such tissues may affect systemic inflammation through the release of a pro-inflammatory cocktail of cytokines and chemokines. Visceral fat tissues are particularly involved in metabolic defects during obesity [4], and inflammation in visceral fat may drive part of the metabolic syndrome [5], [6]. Besides affecting whole body metabolism, inflammation in visceral fat tissue is also commonly associated with Crohn's Disease, a chronic inflammatory affliction of the gastro-intestinal tract [7], [8]. Given the strong association of visceral fat inflammation with metabolic disorders and with inflammatory bowel diseases, it is of crucial importance to elucidate how and why expanding adipose tissues become inflamed.

Studies with mice on high-fat diets have demonstrated that expanding adipose tissues become infiltrated with macrophages, which may be responsible for most of the inflammatory events in these tissues [9], [10]. However, the infiltration of macrophages is a relatively late event in diet-induced obesity, and their accumulation is preceded by the accumulation of T-lymphocytes [11]. These infiltrating cells may play an important role in the recruitment of macrophages [12] and in the regulation of the inflammatory response [13], [14].

Whereas the discovery of the involvement of lymphocytes, macrophages, and other immune cells, such as mast cells [15], in adipose tissue inflammation in obesity has led to an increasingly detailed description of adipose tissue inflammation, it is still not clear why leukocytes are attracted. The focus of the quest for causal factors has mainly been on endogenous or dietary factors, such as saturated fatty acids [16], [17]. However, it is now clear that the intestinal microflora is of pivotal importance in obesity and metabolic syndrome [18], [19], [20], perhaps by affecting dietary energy harvest [18]. Another possibility is that high fat diets, which exert important stress to the intestinal epithelium [21], promote the intestinal absorption of antigenic material from the gut, which then could induce inflammatory immune responses, especially in tissues in close proximity to the gut. We have previously demonstrated that intestinal absorption of dietary fat promotes the absorption of gut-derived lipopolysaccharides (LPS) and of a protein antigen (ovalbumin; OVA), and both were significantly associated with chylomicrons [22], [23]. Since chylomicrons are cleared in part in adipose tissue [24], [25], we tested, in the present study, whether fat absorption also promotes OVA absorption into adipose tissues, and whether this can promote T-lymphocyte responses and inflammation.

We observed that fat absorption indeed promoted antigen absorption into adipose tissue. Moreover, mice previously sensitized to the antigen showed significant inflammatory responses in mesenteric, but not subcutaneous, adipose tissues, and these responses were further enhanced during high-fat dieting. Over time, these responses resulted in a decrease of glucose tolerance. We propose that intestinal antigen absorption may be a contributor to inflammation of visceral adipose tissue during high-fat feeding.

## Materials and Methods

### Materials, reagents

Medium-chain triglyceride (MCT) oil was from Novartis, the long-chain triglyceride (LCT) consisted of food grade soybean oil. Intralipid (20%) was from Glaxo-Welcome. Pluronic L-81, a surfactant blocking chylomicron secretion from enterocytes at pharmacological dose [26], was a generous gift from BASF corporation (Florham Park, NJ). Diets were custom prepared by Research Diets Inc., based on the D12450B low-fat (10 kcal% fat) or D12492 high-fat (60 kcal% Fat) diets, and were modified by exchanging 5% of the dietary protein with egg white for a final OVA content of 1% by weight. Anti-CD3 was from Abcam (ab5690), and was visualized in immunohistochemistry with Alexa-Red 568-labeled goat anti-rabbit IgG from Invitrogen (A-11011). The anti-osteopontin antibody was from R&D Systems (AF808) and was visualized by diaminobenzamidine staining using an anti-goat ABC kit from Pierce. Alexa-488, Alexa-647, and PE-labeled anti-mouse CD3, CD4, and CD8 antibodies and their isotype controls were from BioLegend. Ovalbumin (Sigma-Aldrich Grade V) was radiolabeled with ^125^-I as described elsewhere [22].

### Mice

Male BALB/C mice and C57Bl/6 mice, ordered at 6 weeks of age from The Jackson Laboratory, were held in a room of a specific pathogen-free animal facility with a 12 h light/dark cycle, and were used at 8 weeks of age. For OVA absorption experiments, mice were fasted (4 h), then gavaged with ^125^I-OVA in various vehicles (0.2 ml). Tissues were harvested from humanely killed mice, after cardiac perfusion with 10 ml cold phosphate-buffered saline (PBS; except for immunohistochemistry experiments). For feeding experiments, OVA-naïve mice were sensitized by two intraperitoneal injections with 10 µg OVA in 0.2 ml alum (Accurate Chemical and Scientific Corp.), with one week between injection. Control mice were injected with alum only. Mice received OVA-containing diets one week after the second injection for indicated durations. For glucose tolerance tests, mice were fasted (4 h) before being intraperitoneally injected with 2 g glucose in PBS/kg bodyweight. Blood samples were obtained from the tail vein at several time points and were tested for glucose concentration with a TrueTrack glucose meter from Home Diagnostics Inc. Adiposity was measured using a EchoMRI-5000 Whole Body Composition machine (Echo Medical System, Houston, TX). All animals were handled in strict accordance with good animal practice as defined by the relevant national and local animal welfare bodies, and all animal work was approved by the Institutional Animal Care and Use Committee of the University of Kentucky (Animal Welfare Assurance Number of the University of Kentucky A3336-01; U.K. IACUC protocol 2008-0306).

### Immunohistochemistry

Adipose tissue samples, isolated while strictly avoiding lymph node material, were fixed in 10% formalin, embedded in paraffin, and cut in 5 µm sections. Antigens were retrieved in deparaffinized and rehydrated sections by boiling in citrate buffer (10 mM, pH 6) for 30 minutes. CD3 was stained by successive incubation with anti-CD3 and Alexa-568 labeled secondary antibody, whereupon the slides were mounted in 4′,6-diamidino-2-phenylindole- (DAPI) containing mounting medium (Vectashield). The slides were observed with an Olympus BX51 fluorescence microscope equipped with a digital camera. Osteopontin (OPN) was detected by using a chromogenic substrate for the secondary antibody according to the kit manufacturer's instructions.

### Flow Cytometry

Stromal vascular cells were isolated from adipose tissue by collagenase digestion as described elsewhere [27]. Fluorescently labeled antibodies, optimized for concentration and specificity using isotype controls, were added to the cells, and these were analyzed, after washing, with a FACScalibur flow cytometer (Becton Dickinson) in the Microbiology, Immunology and Molecular Genetics Core facility of the University of Kentucky. Histograms represent cells gated for lymphocyte phenotype based on forward and side scatter data. Results were plotted with GateLogic software, version 305.

### Quantitative real-time polymerase chain reactions

RNA from adipose tissue samples was extracted with the Trizol-reagent based method. RNA was transcribed into cDNA with the iScript kit from Quanta Biosciences, and the resulting cDNA was amplified using Quanta Bioscience's Perfect Sybr mix using a Biorad iQ5 multicycler. Primer pairs were, in 5′-3′ direction, AGC-AAG-AAA-CTC-TTC-CAA-GCA-A/GTG-AGA-TTC-GTC-AGA-TTC-ATC-CG (OPN), TTG-GCC-AGC-GCC-ATC-TT/CCT-GTT-GCT-GTA-GCC-GTA-TTC-A (Glyceraldehyde 3-phosphate dehydrogenase (GAPDH)), ATG AAC GCT ACA CAC TGC ATC/CCATCC TTT TGC CAG TTC CTC (Interferon gamma (IFNγ)), and TTG GCC AGC GCC ATC TT/TGC CTC CTC CAG AGA GAA GTG (Forkhead Box P3 (FOXP3)).

### Statistical Analyses

Comparison of gene expression between naïve or sensitized mice on low- or high-fat OVA diets was performed with two-way ANOVA and Bonferroni's post-hoc tests using XLstat Software (Addinsoft SARL). Plasma glucose levels over time were compared between groups with a linear mixed model using SAS software. OVA absorption data were tested for statistical significance by ANOVA with a Bonferroni post-hoc test, and T-lymphocyte subtype percentages with Student's T-test, using Graphpad Prism v5. All bar graphs represent average values ± S.D.

## Results

### Adipose tissue contains intestinally absorbed antigen (OVA)

**Figure 1:**
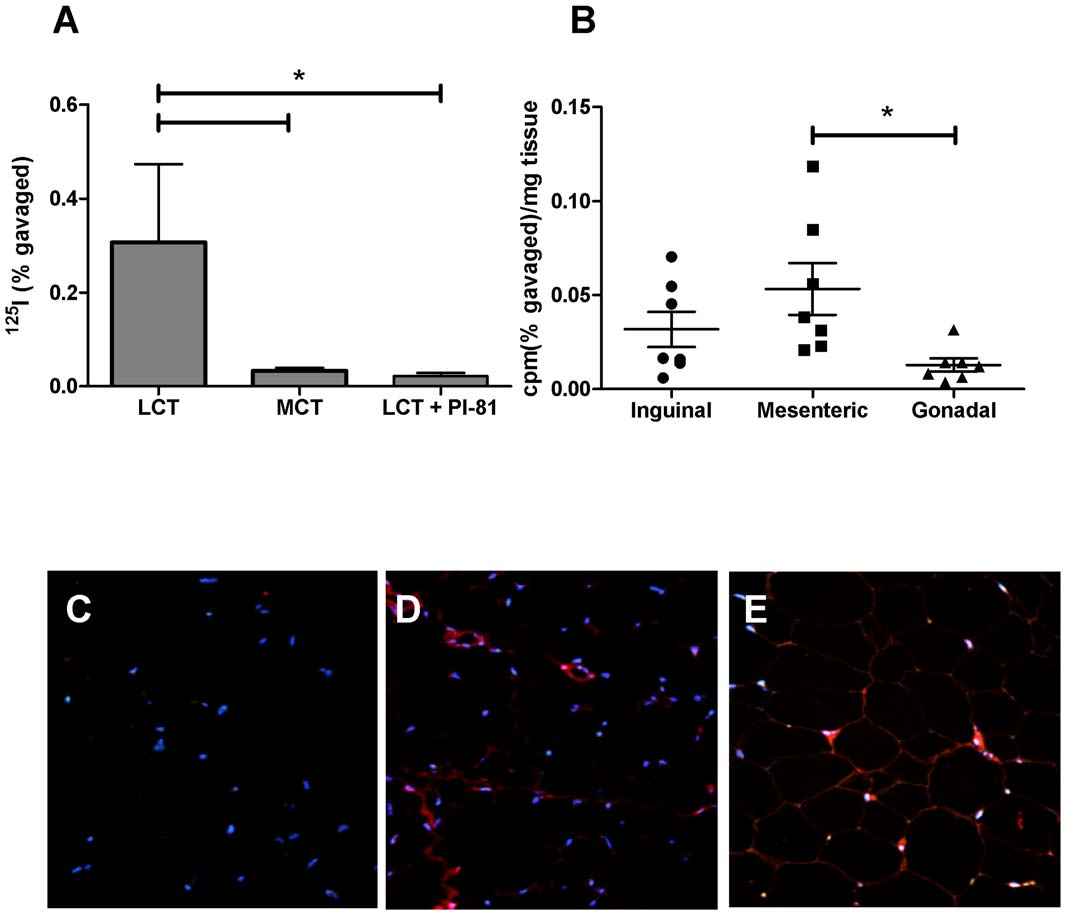
Intestinal absorption of dietary antigen (OVA) into adipose tissue. (A) Fasted BALB/c mice (n = 4) were gavaged with 0.2 ml LCT, MCT, or LCT plus the inhibitor of chylomicron formation Pluronic L-81, and identical amounts of ^125^I-OVA. Gonadal adipose tissue was removed 60 minutes later and radioactivity was measured and normalized to tissue weight. (B) shows appearance of ^125^I-OVA into indicated adipose tissue samples 15 minutes after gavage of ^125^I-OVA in 0.2 ml 20% Intralipid. Asterisks indicate statistically significant differences (P<0.05) by one-way ANOVA with Bonferroni-adjusted post-hoc tests. (C–E) OVA immunostaining (red signal) in mesenteric adipose tissue of mice on OVA-free diets (C) or 1% OVA diets with low- (D) or high- (E) fat content. Blue signals represent DAPI-stained nuclei. OVA is mainly detectable in apparent endothelial cells, but staining can also be observed in other SVF cells and adipocytes.

We have recently demonstrated in experiments with mice that dietary long-chain triglycerides (LCT) significantly enhanced intestinal absorption of concomitantly ingested dietary OVA, and that a significant fraction of the OVA was associated with chylomicrons [22]. To test whether intestinally absorbed OVA reaches adipose tissue, a major site of chylomicron clearance [24], [25], we gavaged fasted mice with ^125^I- OVA in 0.2 ml LCT, MCT (medium-chain triglycerides), or LCT to which the chylomicron secretion inhibitor Pluronic L-81 (Pl-81) was added. We observed that gonadal adipose tissue, isolated 60 minutes after gavage, contained significantly more ^125^I when the OVA was gavaged with LCT compared with MCT or LCT plus Pl-81, suggesting that chylomicron formation, essential for dietary fat absorption, promotes absorption of gut antigen into adipose tissue (Figure 1A).

Next, we attempted to determine the preferential adipose tissue target for absorbed antigen. Fasted mice received an intragastric bolus of ^125^I-OVA in 0.2 ml of 20% Intralipid, and ^125^I levels were measured in mesenteric (visceral), gonadal (visceral), and inguinal (subcutaneous) adipose tissue fifteen minutes after gavage. Pilot experiments had revealed similar levels of radioactivity in the tissues 15 minutes and 1 h after gavage. Gonadal adipose tissue contained the least ^125^I, and mesenteric adipose tissue the most, although the difference between mesenteric and subcutaneous fat did not reach statistical significance (Figure 1B).

We next tested whether the radiolabel in adipose tissue represented antigenic OVA and not just ^125^I-breakdown products. This was done by immunohistochemistry of mesenteric adipose tissue isolated from OVA-naïve mice fed with 1% egg white- enriched diet for two weeks. We observed substantial OVA staining in the adipose tissue of OVA-fed mice, with most of the signal in cells of the stromal vascular fraction (SVF; Figure 1D, E). Mice on high-fat diets seemed to have more pronounced OVA staining, especially in association with the SVF. The absence of signal in mice on egg-free standard laboratory diets indicated staining specificity. Collectively, these data indicate that a fraction of antigenic material in the gut is absorbed into adipose tissue. The involvement of chylomicronemia in the process suggests that increased fat consumption could lead to increased antigen absorption into adipose tissue.

### T-lymphocytes accumulate in adipose tissue in response to dietary OVA

**Figure 2:**
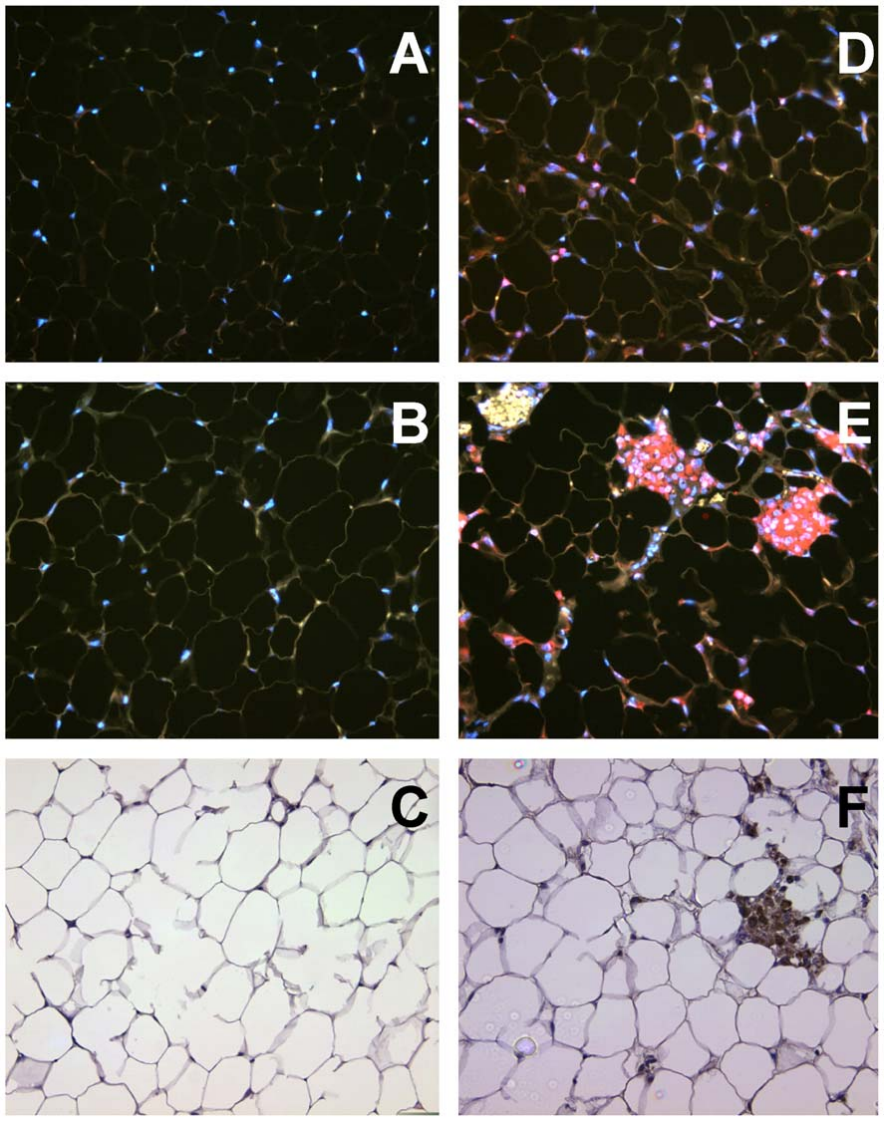
Expression of CD3 and osteopontin in mesenteric fat in response to dietary antigen. Naïve mice (A–C) or OVA-sensitized mice (D–F) were fed 1% egg-white diets for two weeks, and mesenteric adipose tissue was stained for CD3 (Panels A,B, D, E; red signal) or osteopontin (C, F; brown signal). Nuclei in A,B,D and E were stained blue with DAPI.

To test whether gut antigens in adipose tissue can provoke T-lymphocyte responses, we fed OVA-sensitized or OVA-naïve BALB/c mice a diet enriched with 1% egg white for two weeks. Mesenteric adipose tissue samples, isolated after removing the lymph nodes, were embedded in paraffin for sectioning and staining with anti-CD3. Whereas CD3+ cells were barely detectable in mesenteric adipose tissue from OVA-naive mice (Figure 2A,B), such cells were present in the tissue of sensitized mice, either in clusters (Figure 2E) or more dispersed throughout the tissue (Figure 2D). All of the 3 sensitized mice that were thus analyzed, but none of the three naïve mice, contained several CD3+ cell clusters. CD3 immunostaining in OVA-sensitized mice on OVA-free diets was not different from that in naïve mice (not shown).

**Figure 3:**
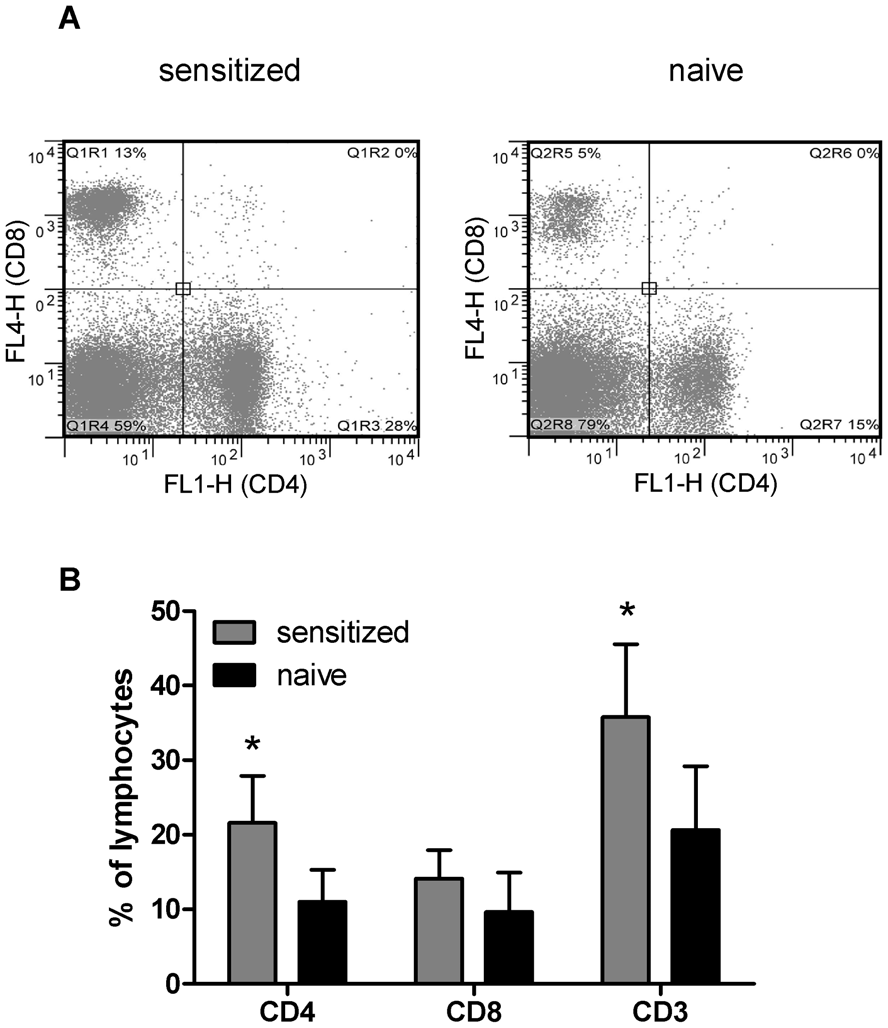
CD4 T-cell accumulation in mesenteric adipose tissue of OVA-fed, OVA sensitized mice. Naïve or OVA-sensitized C57Bl/6 mice were fed 1% OVA-containing high-fat diets for 14 weeks and SVF cells from their mesenteric adipose tissue were analyzed by flow cytometry. Two representative scatter plots are shown (A). The bar graph (B) shows the average percentage CD4 or CD8 T-lymphocytes ± S.D. (n = 5 mice per group). The asterisk (*) indicates statistically significant differences (P<0.05; Student's T-test).

To determine whether the T-lymphocytes were CD4+ or CD8+, we fed naïve or sensitized C57Bl/6 mice with 1% OVA-containing high-fat diets for 14 weeks and isolated the SVF from their mesenteric adipose tissue for flow-cytometry. As shown in Figure 3, the SVF lymphocyte fraction of sensitized mice showed significant increases in the number of CD3+ cells, with the majority being accounted for by CD4 T-lymphocytes. Thus, it appears as if antigen-sensitized mice show increased CD4 T-lymphocyte infiltration into antigen-containing mesenteric adipose tissues.

The apparent T-lymphocyte response to gut antigens prompted us to investigate whether the adipose tissue was inflamed. We used immunohistochemistry to determine whether the apparent T-lymphocyte clusters expressed osteopontin. This pro-inflammatory protein is produced by several cells, including T-lymphocytes (it is also referred to as Early T Lymphocyte Activation 1; Eta-1), and has been implicated in adipose tissue inflammation in diet-induced obesity [27]. Similar to our findings with CD3, we did not detect osteopontin-expressing cells in adipose tissue from OVA-fed, OVA-naïve mice (Figure 2C). In contrast, the clusters of apparent T-lymphocytes present in mesenteric adipose tissue of OVA-fed, OVA-sensitized mice showed considerable reactivity with anti- osteopontin-antibodies (Figure 2F). Thus, the apparent accumulation of T-lymphocytes in mesenteric adipose tissue was associated with increased expression of pro-inflammatory osteopontin.

### Increased inflammatory gene expression in mesenteric, but not subcutaneous adipose tissue in OVA-sensitized OVA-fed mice

**Figure 4:**
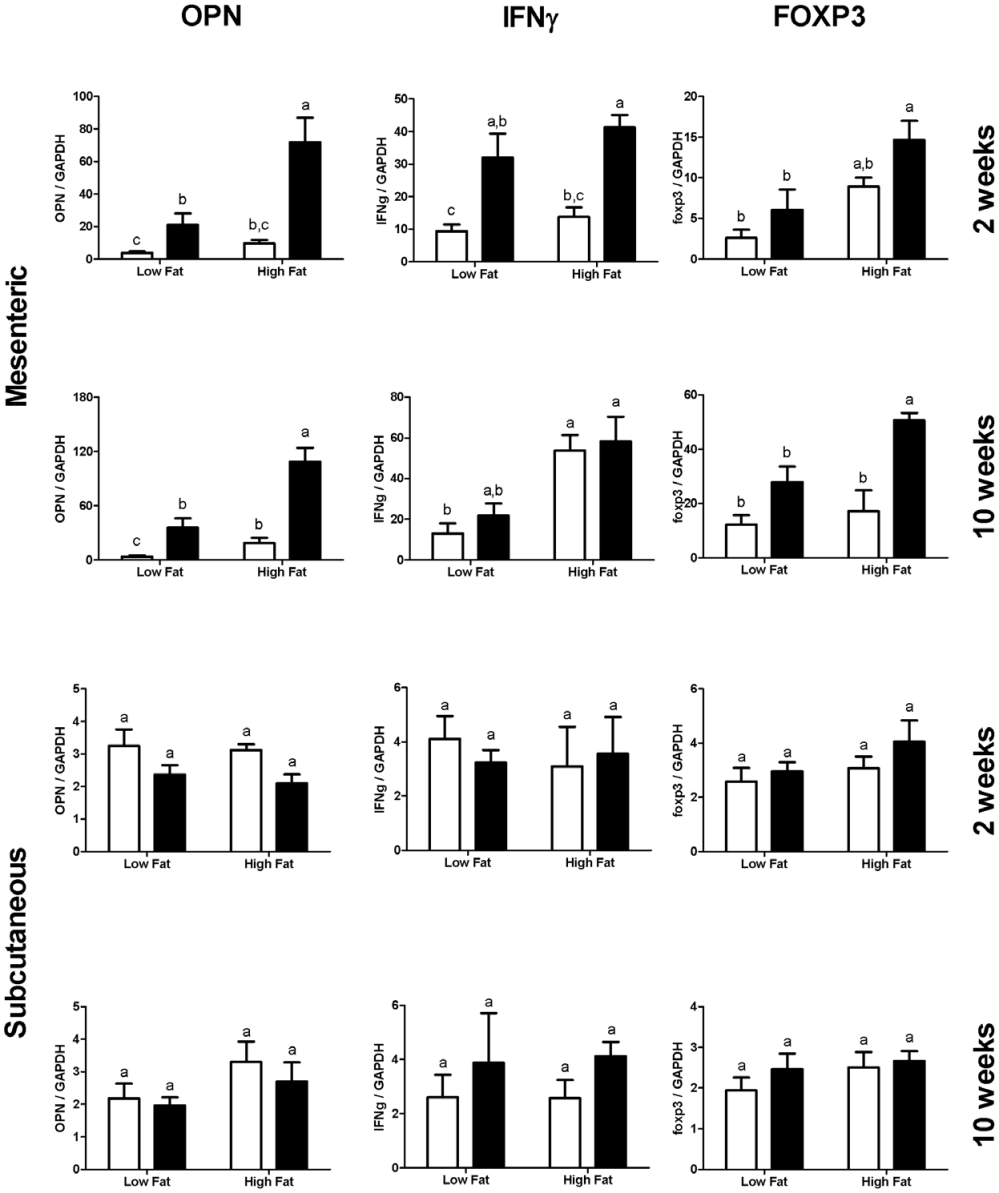
Inflammatory gene expression in mesenteric and subcutaneous fat in response to gut antigen. OVA-sensitized BALB/c mice (black bars) or naïve BALB/c mice (white bars) were fed 1% egg-white-containing low- or high- fat diets for 2 or 10 weeks, and expression of OPN, IFNγ and FOXP3 were measured in mesenteric and subcutaneous adipose tissue. Groups contained five to six mice, and were compared by two-way ANOVA after log-transformation of the data. Groups not sharing the same letter were considered statistically significantly different by Bonferroni-adjusted post-hoc tests.

To further investigate the potential contribution of T-lymphocyte responses to intestinally absorbed antigens in adipose tissue inflammation, we fed OVA-naïve or OVA-sensitized mice with low- or high- fat diets containing 1% egg-white powder. Mesenteric and inguinal adipose tissue samples were obtained after two or ten weeks. As expected, osteopontin gene expression increased significantly in mesenteric adipose tissue in mice on high-fat diets. Differences were already apparent after two weeks. However, osteopontin expression in subcutaneous fat was not significantly affected by dietary fat content (Figure 4). These observations are in line with the notion that visceral fat is more readily inflamed in diet-induced obesity than subcutaneous fat [4], [5], [6]. Strikingly, mice responding to the absorbed gut antigen (OVA sensitized mice) showed even higher osteopontin gene expression in their mesenteric fat. Similar results were observed with interferon gamma, another Th1 cytokine implicated in adipose tissue inflammation in diet-induced obesity [28], except that the difference was no longer apparent after 10 weeks. This could be due to the fact that the expression of FOXP3 (Forkhead Box P3), a marker for regulatory T-lymphocytes, was upregulated. This observation confirms that T-lymphocytes accumulate in mesenteric fat (since FOXP3 is restricted to T-lymphocytes) and indicates that mechanisms are mounted to suppress OVA-driven inflammation.

### Effect of antigen-driven inflammatory immune responses in adipose tissue on bodyweight, adiposity, and glucose tolerance

**Figure 5:**
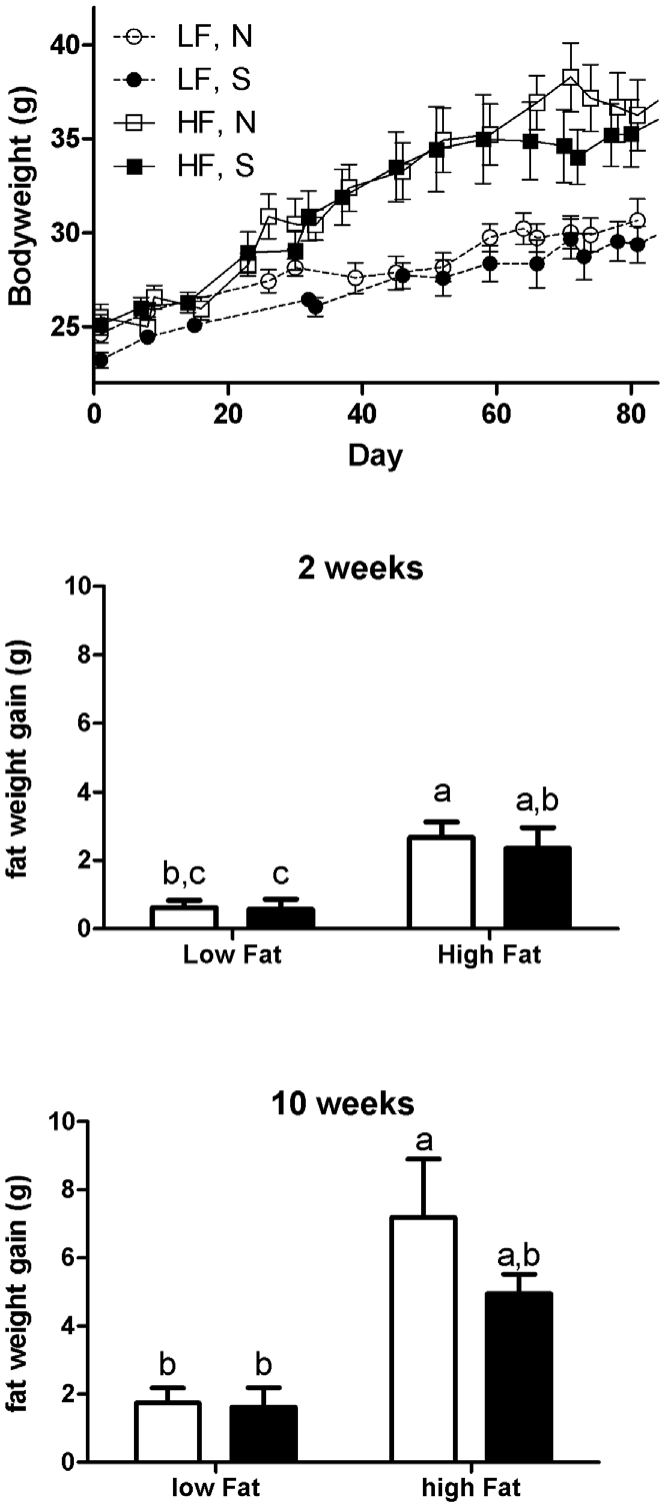
No significant effect of immune responses to gut antigen on body weight or fat mass. Groups of naïve or sensitized BALB/c mice (n = 6 per group) were fed 1% OVA containing low- or high- fat diets. Whereas mice on high-fat diets gained more weight than mice on low-fat diets, there was no significant difference between naïve and sensitized mice within each diet group. LF  =  low-fat diet, HF  =  high fat diet, N =  naïve, S =  sensitized. (B) Weight gain of body fat (in grams) of naïve (white bars) or sensitized (black bars) mice (n = 6 per group) on 1% OVA diets for 2 weeks (B) or 10 weeks (C). Bonferroni-adjusted post-hoc tests following two-way ANOVA revealed statistically significant differences between groups not sharing the same letter.

**Figure 6:**
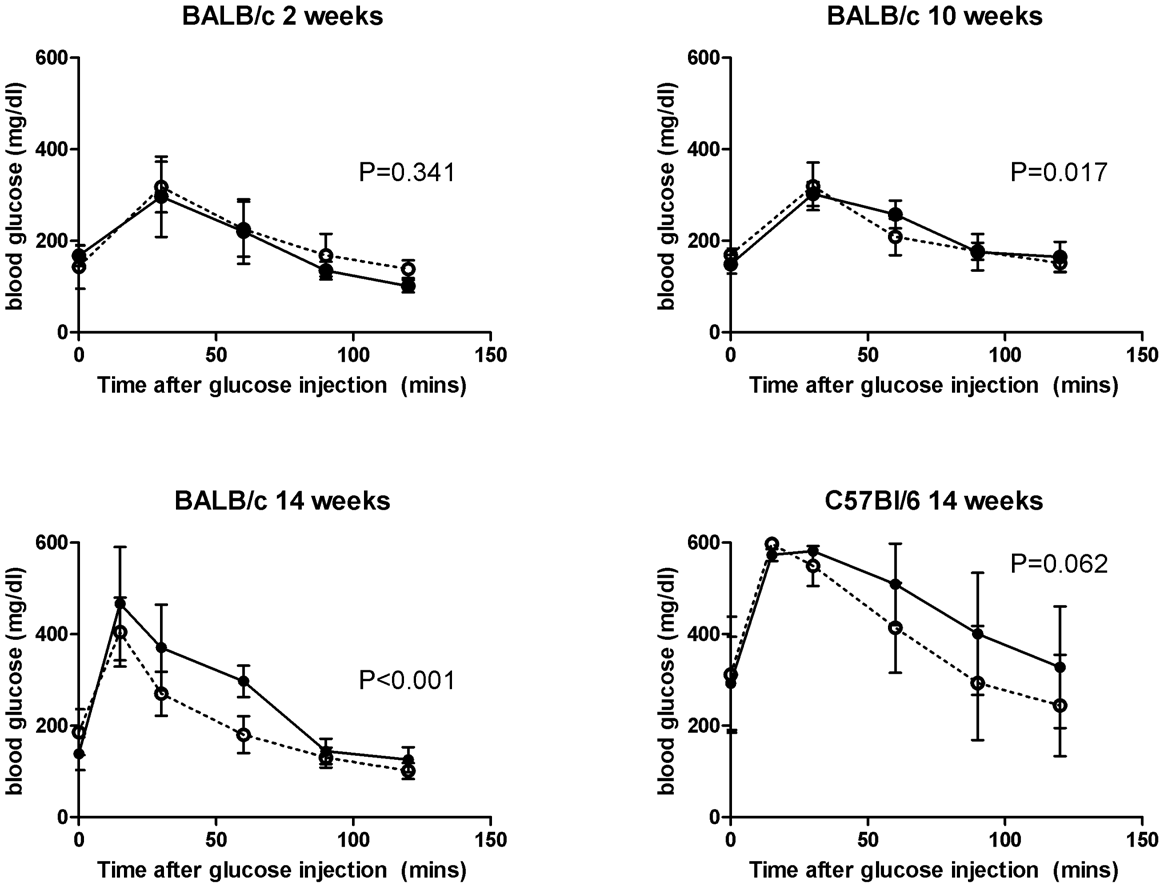
Inflammatory immune responses to gut antigen in mesenteric adipose tissue impair glucose tolerance. OVA-sensitized (solid lines and symbols) or naïve (dashed lines, open symbols) mice were put on 1% OVA-containing high-fat diets for the indicated duration, and a glucose tolerance test was then performed on fasted mice. Glucose clearance was significantly impaired in sensitized BALB/c mice after 10 and 14 weeks (linear mixed model test for identical trajectories) and trended to significantly decrease in C57Bl/6 mice after 14 weeks.

To test the physiological implications of apparent inflammatory immune responses to gut antigens in adipose tissue, we sensitized BALB/c mice with OVA plus alum or with alum only (control) and fed them low or high-fat diets enriched with 1% OVA. Both naïve and sensitized mice (n = 6 per group) showed similar weight gain within their dietary treatment over the course of the experiment, and total fat weight gain was similar at 2 and 10 weeks (Figure 5). However, glucose tolerance tests of mice on OVA-containing high fat diets revealed that sensitized BALB/c mice showed significantly impaired clearance of blood glucose after 10 and 14 weeks (Figure 6B, C). In C57Bl/6 mice, glucose tolerance tended to significantly differ after 14 weeks (Figure 6D). The lack of significance in C57Bl/6 mice may be due to the fact that blood glucose levels exceeded the detection ceiling of the test during the first hour.

## Discussion

Mounting evidence indicates that T-lymphocytes are involved in the early stages of visceral adipose tissue inflammation in diet-induced obesity. However, it is still unclear why T-lymphocytes are attracted. In this study, we provide evidence for a potential role of antigenic material absorbed from the gut in T-lymphocyte activation in visceral adipose tissue. First, we show that intestinal absorption of dietary fat also stimulates the intestinal absorption of a gut antigen, ovalbumin (OVA), into adipose tissue. Second, we show that under conditions of lack of immunological tolerance to such an antigen, mesenteric adipose tissue is a site of T-lymphocyte dependent inflammation. Third, we show that prolonged exposure to an antigenic insult from the gut can lead to a loss of glucose tolerance, an important feature of metabolic syndrome. Taking these observations into consideration, we propose that T-lymphocyte activation in visceral adipose tissue during diet-induced obesity could be, at least in part, a response to influx of antigenic material absorbed from the gut.

Central adiposity is an important component of metabolic syndrome. The problem with expanded visceral fat is that it is frequently inflamed, and this can affect other tissues and lead to deregulation of lipid and carbohydrate metabolism. A hallmark of adipose tissue inflammation in diet-induced obesity is the infiltration of macrophages [9], [10]. However, more recent work has shown that macrophage infiltration occurs relatively late in diet-induced obesity, and is preceded by T-lymphocytes [11]. CD4 and CD8 T-lymphocytes have since been implicated in macrophage recruitment and in the regulation of the inflammatory response in adipose tissue [12], [13], [14]. However, these studies still do not explain why T-lymphocytes are attracted in the first place. Since excess fat intake is a major cause of obesity in Western societies, it has been suggested that excess free fatty acids can cause adipose tissue inflammation through activation of innate immune receptors [16], [17]. However, high-fat diets fail to induce obesity and the associated chronic inflammation in germfree mice [19], [20], [29], [30]. This prompted us to test whether antigen absorption from the gut could elicit inflammatory immune responses in visceral adipose tissue, and whether excess dietary fat promotes such responses.

We and others have previously demonstrated that there is a link between fat absorption and the absorption of bacterial LPS from the gut [23], [31], [32]. Interestingly, systemic infusion of absorbed amounts of LPS in mice was sufficient for diet-induced obesity and metabolic syndrome, even when the mice were fed standard diets [32]. We have observed that LPS absorption largely depends on the formation of chylomicrons, and that these particles contain most of the absorbed LPS [23]. Since chylomicrons transport dietary fat to adipose tissue [24], [25], these tissues are likely exposed to considerable amount of LPS. We recently found that a protein antigen, ovalbumin (OVA), is also increasingly absorbed during chylomicron formation, and is also associated with these particles [22]. This may explain why we observed that chylomicron formation promoted the delivery of dietary OVA to adipose tissues. Immunohistochemistry suggested that the antigen was present in several cell types, including adipocytes and SVF cells, with prominent staining in endothelial cells, which bind chylomicrons during lipolytic fatty acid transfer. The fact that chylomicron formation promotes OVA absorption into adipose tissue suggests that increased fat intake, which leads to increased postprandial chylomicronemia, leads to increased antigen exposure of adipose tissue.

The apparent absorption of an antigen and its uptake into tissues does not necessarily mean that it will cause inflammatory immune responses. In fact, naïve mice typically develop oral tolerance to absorbed antigens, which protects against allergic or inflammatory responses [33]. Indeed, when we fed naïve mice (i.e., mice that were injected intraperitoneally with OVA-free adjuvant) with OVA-enriched diets, we did not observe T-lymphocyte infiltration into adipose tissue even though antigen was present. However, when we sensitized the mice before the feeding started, we observed significant CD4 T-lymphocyte infiltration into the tissue. This was associated with an antigen-specific increase in inflammatory gene expression of mesenteric adipose tissue. In the early stages of antigen feeding (2 weeks), the apparent inflammatory responses in the adipose tissue did not significantly affect body weight gain or adiposity, and did also not affect the clearance of blood glucose. However, after 10 and 14 weeks, the mice displayed a significant loss of glucose tolerance. The fact that it took so long for glucose intolerance to occur could perhaps be due to the concomitant increase in FOXP3 expression in adipose tissue, which indicates that regulatory T-lymphocytes are present, likely in an attempt to suppress inflammation.

Our model could be seen as a rather artificial situation, with an antigen not known to affect obesity and metabolic syndrome and with an artificially induced immunological sensitivity to the antigen. Moreover, the mice were sensitized intraperitoneally, which may have biased the response somehow to the viscera. Nevertheless, we clearly observed that even OVA-naïve mice showed significant inflammation in mesenteric adipose tissue during high-fat feeding, in contrast to subcutaneous adipose tissue, which was refractive. We speculate that this difference is due to increased exposure of mesenteric fat to antigenic material from the gut. We believe that our work is novel in that it shows, for the first time (as far as we know), that antigenic material can be absorbed from the intestine into adipose tissue, and that high-fat diets and such antigens mutually reinforce mesenteric adipose tissue inflammation. Since our intestines contain large amounts of microbial antigens with inflammatory potential, and since their presence is required for high-fat diet-induced obesity and metabolic syndrome [19], [20], [30], it is tempting to speculate that such antigens can play a role in visceral adipose tissue inflammation. Antigen absorption and systemic antigen dissemination certainly do not restrict the antigen to adipose tissue, even when the antigen is associated with chylomicrons [22]. We observed substantial antigen accumulation in other tissues, such as the liver and skeletal muscle. When we looked at the liver, we found that sensitized mice showed increased OPN expression during OVA-feeding (unpublished observations). It is possible that inappropriate responses to gut antigens (perhaps from the microflora) have deleterious consequences for many physiological processes.

In summary, we propose a novel mechanism for T-lymphocyte dependent mesenteric adipose tissue inflammation in obesity: High-fat diets promote intestinal absorption of gut antigens, and the delivery of the antigens to adipose tissues, preferentially visceral depots. This leads to early infiltration of T-lymphocytes into the tissue. A pro-inflammatory milieu within the visceral fat, perhaps through increased free fatty acids, might increase the risk for loss of tolerance to the antigens, favoring chronic inflammation.
